# What Drives Pet Food Choices? A Systematic Literature Review

**DOI:** 10.3390/ani15223235

**Published:** 2025-11-07

**Authors:** Chen Ai, Faical Akaichi, Klaus Glenk, Cesar Revoredo-Giha, Montserrat Costa-Font

**Affiliations:** School of Natural and Social Sciences, Scotland’s Rural College, West Mains Road, Edinburgh EH9 3JG, UK; faical.akaichi@sruc.ac.uk (F.A.); klaus.glenk@sruc.ac.uk (K.G.); cesar.revoredo@sruc.ac.uk (C.R.-G.); montse.costafont@sruc.ac.uk (M.C.-F.)

**Keywords:** pet food, pet owners, preferences, attitudes, drivers

## Abstract

**Simple Summary:**

The global pet population has increased significantly over the last decade. As with the growing pet population, the pet food industry is also growing rapidly worldwide. Although new products continue to be developed and introduced in pet food markets every year, insights into consumer behaviour towards pet food products have remained largely underexplored. This review aims to summarise the various drivers that affect pet owners’ purchase decisions of pet food products. Intrinsic and extrinsic product characteristics, psychological, biological and physiological, socio-cultural, and situational factors were identified as the key factors driving pet owners’ pet food purchase decisions. This work reveals a highly fragmented nature of the state-of-the-art in the field of consumer behaviours towards commercial pet food products, with most studies focusing on only several aspects of the drivers. It also provides the gaps in the extant literature and potential research directions for future consumer behavioural research in this domain.

**Abstract:**

The rapid expansion of the global pet food industry has intensified interest in understanding the factors shaping pet owners’ purchasing decisions. This systematic literature review synthesises evidence from 40 peer-reviewed studies published between 2006 and 2024 to identify the key drivers of consumer behaviour toward commercial pet food. Following PRISMA guidelines, articles were retrieved from Scopus, Web of Science, and Google Scholar. Findings reveal six main categories influencing purchasing behaviour: intrinsic and extrinsic product characteristics, psychological, biological and physiological, sociocultural, and situational factors. Product quality, ingredient composition, price, brand reputation, sustainability, and pet health status emerged as consistent determinants of choice. The review highlights that consumer behaviour toward pet food remains a developing research field with limited cross-regional studies and methodological diversity. Future research should expand geographically and explore the intersection of sustainability, pet welfare, and owner psychology in pet food decision-making.

## 1. Introduction

As of 2024, it is estimated that half of the global population owns at least one pet. The United States of America (USA) leads with around 70 million dogs and 74 million cats, followed by China, which has 27 million dogs and 53 million cats [1]. In the European Union (EU), Germany and the United Kingdom (UK) are the top pet-owning countries, with Germany having over 10 million dogs and 15 million cats and the UK with approximately 13 million dogs and 12 million cats [1]. In South America, Brazil records the largest number of dogs—around 35 million—making it the country with the second-largest dog population in the world [1].

The rise in global pet ownership has led to significant growth in the pet food market, reaching a revenue of 151.10 billion dollars in 2024, with an anticipated annual growth rate of 5.26% from 2024 to 2029 [2]. The USA accounts for over one-third of the global pet food market revenue, generating 59.74 billion dollars in 2024. China ranks second with 8.2 billion dollars, followed by the UK and Brazil, with revenues of 7.46 billion dollars and 6.88 billion dollars, respectively [2].

Distribution channels for pet food are also evolving, with online shopping accounting for approximately 37% of total pet food revenue in 2024 [2]. This growth is projected to continue driven by the recent surge in direct-to-consumer channels [3], appealing to Generation Z (Gen Z) and Millennials who value convenience and increasingly use online subscription services [3]. Meanwhile, brick-and-mortar pet retailers and brands are transforming to attract customers and seize market opportunities. For example, JustFoodForDogs operates 11 standalone, open-to-the-public retail kitchens nationwide, allowing customers to observe its operations and the entire food preparation process [4]. Driven by the rise in demand for pet food, the industry has stimulated new product development, raising the pet food offer to more than 3000 options for pet owners to choose from [5].

Pet ownership has been associated with a range of potential health benefits. Studies suggest that having a pet may help lower the risk of systemic hypertension [6,7] and hyperlipidaemia [8], decrease the likelihood of cardiovascular disease [9] and reduce the frequency of doctor visits, leading to lower healthcare expenses [10]. Additionally, pet owners were found to exhibit higher levels of physical activity [11,12,13], a lower prevalence of obesity [14,15,16], and improved mental well-being [17,18,19,20].

Mirroring trends observed in the human food system, the growing number of pet owners and the increasing demand for pet food have given rise to two major concerns: the rising prevalence of overnutrition among pets and the escalating environmental footprint of pet ownership. The 2022 U.S. Pet Obesity Prevalence Survey revealed that 61% of cats and 59% of dogs were overweight or obese [21]. Similarly, Montoya et al. [22] reported that 26% of dogs and 40% of cats in the USA had overweight or obese body conditions during young adulthood. High rates of pet obesity have also been documented in the UK. A 2023 survey commissioned by UK Pet Food, which included 148 veterinary professionals and 2558 households, found that 50% of dogs, 43% of cats, 31% of small mammals, and 9% of birds were overweight [23]. Despite veterinarians unanimously expressing concern over this growing issue, only 4% of pet owners surveyed recognized it as a problem [23].

Obesity in dogs and cats poses a significant health concern due to its strong association with multiple diseases. In both species, excess body fat increases the risk of developing diabetes mellitus [24,25], cardiovascular alterations [26], musculoskeletal and orthopaedic disorders such as arthritis [27,28], urinary tract diseases [25,29], and respiratory issues such as brachycephalic obstructive airway syndrome in dogs and feline asthma in cats [25,30]. Excess body weight also shortened life expectancy in dogs [31] and cats [32].

Alongside the growing concern over pet overnutrition and its adverse health outcomes, recent research has increasingly highlighted the significant environmental footprint of pet food across its entire supply chain—from manufacturing to consumption [33]. For instance, Su and Martens [34] estimated that the diets of Japan’s dog and cat population produce between 2.5 and 10.7 million tons of greenhouse gases (GHGs) each year. Likewise, Yavor et al. [35] found that an average-sized dog generates a lifetime carbon footprint of about 8200 kg CO_2_-equivalent—approximately 7% of the annual emissions of an average EU citizen. In the USA, Okin [36] reported that the country’s 78 million dogs and 86 million cats account for 25–30% of the environmental impact from animal production and up to one-quarter of livestock-related GHG emissions, which remain the largest source of agricultural emissions globally. More recently, Alexander et al. [33] estimated that global pet food production contributes approximately 56–151 million tonnes of CO_2_-equivalent emissions—representing 1.1–2.9% of total agricultural emissions. It also accounts for an estimated 41–58 million hectares of agricultural land use (0.8–1.2% of the global total) and 5–11 cubic kilometres of freshwater consumption (0.2–0.4% of total agricultural use). The researchers concluded that pet food production should be recognized as a significant component of the global food system and incorporated into broader discussions and research on food sustainability.

Addressing the growing health and environmental challenges linked to pet food systems is essential for ensuring animal well-being and planetary sustainability. The rising prevalence of obesity among pets and the substantial ecological footprint of pet food production—ranging from greenhouse gas emissions to land and water use—underscore the urgency of action. However, meaningful progress can only be achieved by identifying the underlying causes of these issues and accurately quantifying their impacts. A clearer understanding of these factors will enable the development of more sustainable, health-conscious, and evidence-based strategies for the future of pet nutrition.

To address these research gaps, a comprehensive review of existing studies on commercial pet food purchasing behaviour is essential. To the best of our knowledge, this paper presents the first systematic literature review (SLR) that comprehensively examines the factors influencing pet owners’ purchasing decisions and behaviours toward commercial pet food. This SLR seeks to consolidate current knowledge on pet owners’ purchasing motivations, decision-making processes, and behavioural patterns toward commercial pet foods across multiple disciplines, to identify the key factors that shape these choices. Additionally, by examining geographical focus and methodological approaches of previous research, this review seeks to identify existing gaps and propose clear directions for future investigation.

Specifically, the study addresses the following research questions: (i) What factors influence pet owners’ purchasing decisions and behaviours toward commercial pet foods? (ii) How do pet owners’ purchasing behaviours differ across various types of pet food?

The subsequent sections of this paper are organised as follows: Section 2 presents the methodology, scope, and a summary of the SLR. Section 3 presents the results categorized by thematic areas. Section 4 discusses the findings and identifies research gaps potential research questions for future studies. Section 5 concludes by summarizing the main study’s contributions.

## 2. Materials and Methods

The present study employs the SLR methodology [37]. Following Page et al. [38], the review process included: (i) defining the research scope and objectives; (ii) developing research protocols; (iii) selecting the literature databases; (iv) identifying search keywords; (v) screening eligible articles; and finally (vi) analysing the search results.

### 2.1. Search Strategy

Following Zhang et al. [39], the research started by selecting databases and identifying the relevant search keywords. After an iterative process of searching and refining the keywords, the search concluded when no additional studies met the established eligibility criteria. The selected articles were subsequently organised into an Excel dataset for further analysis. The study search was initiated and limited to 31 December 2024.

#### 2.1.1. Data Source

The present study employed Scopus and Web of Science as the primary search engines in line with a recent SLR paper by Rozenkowska [40]. Both databases can provide extensive access to peer-reviewed journal articles and encompass a wide range of research paper relevant to consumer behaviour research [40]. Google Scholar was used as a supplementary platform for forward and backward searches to improve search robustness and minimise the risk of omitting key articles.

#### 2.1.2. Search Keywords

To identify the most effective keywords for exploring consumers behaviour towards commercial pet food, the search was initially conducted using a broad prompt: consumer behaviour and commercial pet food. This brought limited articles but helped to identify additional keywords. The search was then expanded to include broader keywords, for example, “pet food”, “dog food”, “cat food”, “pet fish food”, “pet rabbit food”, “pet bird food”, “commercial dog food”, “commercial cat food”, “pet owner perceptions”, “pet food choice”, “pet owner purchase intention”, “consumer buying behaviour”, “demand for pet food” etc. After three iterative rounds for refining and updating keywords, the final set were determined and formulated into a comprehensive search string using Boolean logic (“AND”, “OR”). The final list of keywords and the corresponding search string are presented in Table 1.

### 2.2. Eligibility Criteria

Inclusion and exclusion criteria are critical components of high-quality SLR papers. In the present study, the eligibility criteria were developed following the PRISMA approach [39,41] and are outlined in Table 2.

To ensure the quality, readability and availability of articles, only peer-reviewed journal articles or book chapters and technical reports published online and written in English, were selected. To discern potentially predatory or controversial journals. Our evaluation considered several indicators, including absence from major academic indexes (Scopus, Web of Science), lack of transparent peer-review policies, questionable editorial or fee structures, and presence on Beall’s List (archived) or similar sources.

In addition, since some types of pet foods overlap with human food, such as table scrap, home-made food and raw meat, the study only included articles that focus exclusively or partially on commercial pet food to distinguish commercial pet food markets from human food markets. Furthermore, as the present study mainly focuses on consumer buying behaviour towards commercial pet food, only the articles featuring consumer behaviour research were considered.

### 2.3. Search Results

#### 2.3.1. Selected Articles Characterisation

**Figure 1:**
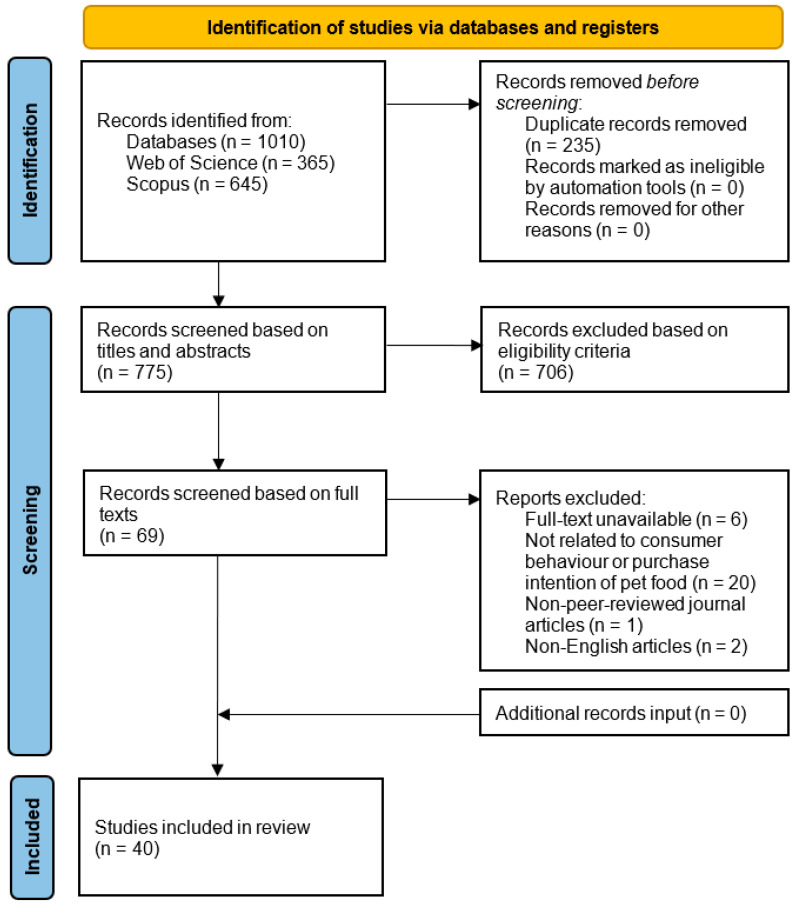
PRISMA flow diagram for the systematic literature review.

1010 records were generated, comprising 365 articles from Web of Science and 645 from Scopus. Prior to the article screening process, 235 duplicates were removed using Excel, leaving 775 records to be screened. The screening was conducted in two phases: first, a qualification assessment based on the title and abstract, and second, a full-text assessment against the eligibility criteria. In the initial screening phase, 706 records were excluded after reviewing the title and abstract, as they were irrelevant to the research topic. This resulted in 69 records being retained for the full-text assessment. In the second phase, a total of 29 records were excluded because they were unavailable online (6 articles), not related to consumer behaviour research on commercial pet food (20 articles), non-peer-reviewed conference papers (1 article), or not written in English (2 articles). Following these removals, 40 articles were retained for the final analysis. The search then used the same search string in Google Scholar to check for missing articles. The first 300 results were analysed. However, no additional articles were identified that met the established eligibility criteria. This resulted in a final selection of 40 articles for further evaluation. The complete PRISMA process is illustrated in Figure 1.

The 40 peer-reviewed articles were published across 24 journals (Table 3). Among these journals, *Animals* accounted for the highest number of articles (8 articles), followed by *PLoS ONE* (4 articles), *Preventive Veterinary Medicine* (3 articles), and *Frontiers in Veterinary Science*, *Journal of Food Products Marketing*, *Journal of Animal Physiology and Animal Nutrition*, and *Journal of the American Veterinary Medical Association*, each with two articles. The remaining 17 journals published only one article each, illustrating the diversity of the literature in this field. Furthermore, the selected articles span a wide range of scientific disciplines, including agricultural and biological science, veterinary sciences, engineering, environmental science, and social sciences. This reflects the interdisciplinary nature of research in this area.

**Figure 2:**
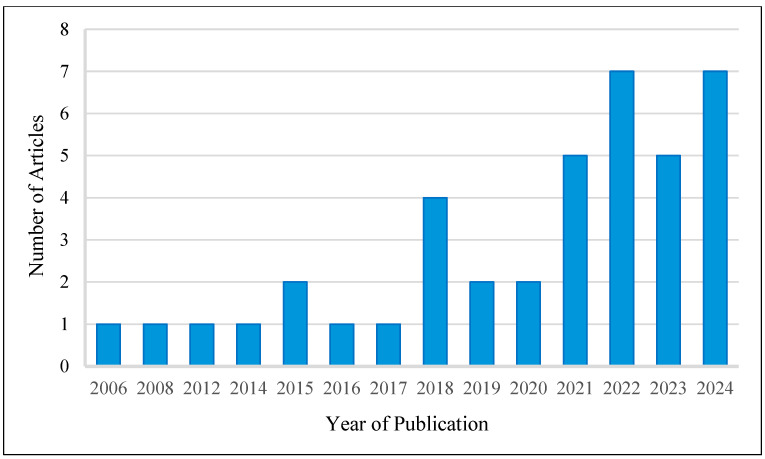
Number of articles published by year.

The temporal distribution of the published articles is presented in Figure 2. The data reveals a noticeable rise in the number of articles in 2018 and from 2020 to 2022, despite the first article being published in 2006. This trend indicates that consumer behaviour towards commercial pet food has recently gained traction as a research topic for scholars, as it has only sparked broader discussions in the last seven years. There is a significant decline in articles published between 2019 and 2020, likely attributable to the disruptions caused by the COVID-19 pandemic. However, the number of articles rapidly increased in the post-pandemic years. In 2024, 7 articles were published, showing sustained and growing interest in this emerging research area.

**Figure 3:**
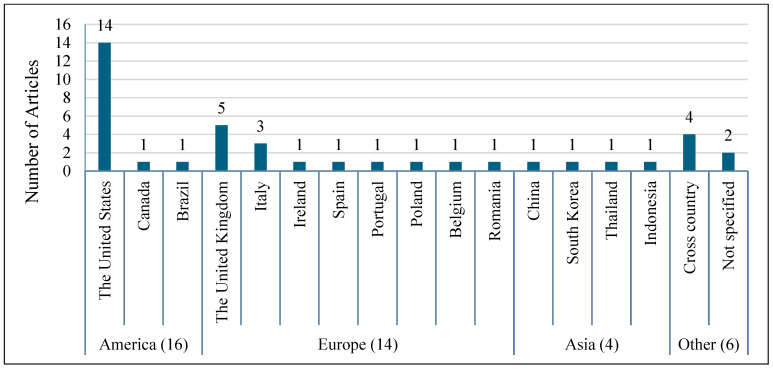
Countries where the studies were conducted.

Consumer behaviour towards commercial pet food is an emerging research area that has garnered significant attention, particularly in developed countries. Of the 40 articles considered in the review, 16 originated from the Americas, predominantly the United States (14 articles), with Canada and Brazil contributing one article each. Europe also experienced a growing body of research in the field of demand for commercial pet food, accounting for 14 articles, including those from the UK (5 articles) and Italy (3 articles). Additional European contributions came from Ireland, Spain, Portugal, Belgium, Poland and Romania, each represented by a single article. In contrast, Asia has a more limited representation despite the high and growing number of pets, with only four articles: China (1 articles), South Korea (1 article), Thailand (1 article), and Indonesia (1 article). Additionally, four articles focused on cross-country data collection, including combinations such as the USA and Canada [54], the USA, Canada, France, the UK and Germany [52], Australia, Canada, Finland, New Zealand, the UK and the USA [53], and the United States and Australia [61]. No studies have been conducted in Africa or exclusively within Oceania. Table 3 summarises the distribution of the articles by country, while Figure 3 presents their continental representation.

#### 2.3.2. Research Methodologies Applied

**Figure 4:**
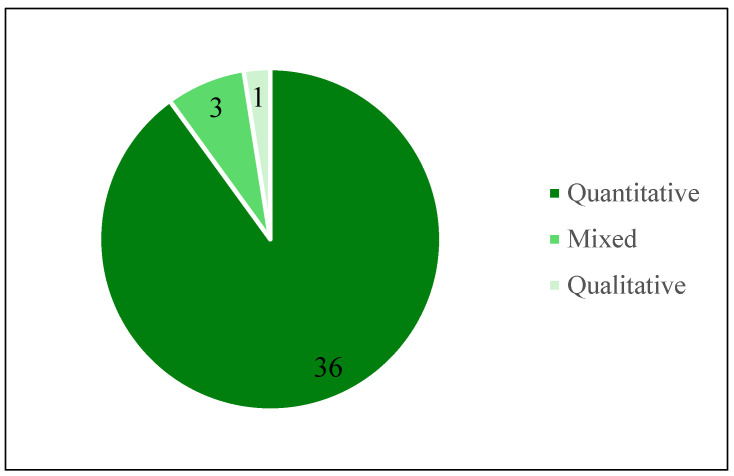
Research methods used in the reviewed articles.

**Figure 5:**
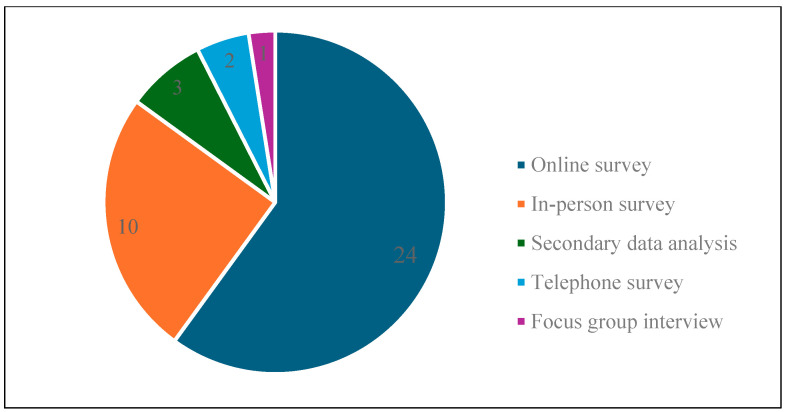
Data collection methods used in the reviewed articles.

**Figure 6:**
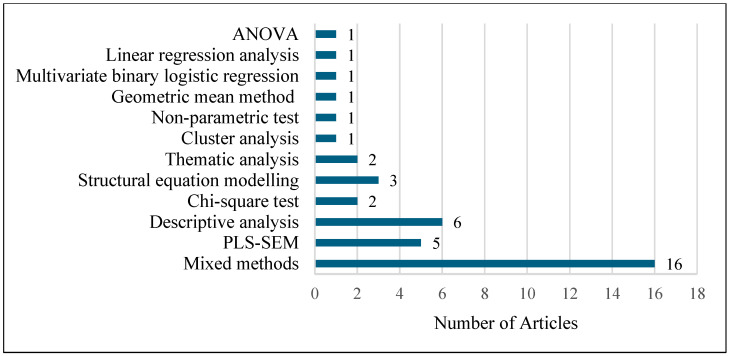
Data analysis methods used in the reviewed articles.

Figure 4, Figure 5 and Figure 6 provide an overview of the types of research methods, data collection techniques, and analysis approaches used in the reviewed studies, respectively. Among the 40 articles examined, quantitative research was the most used type of research, featuring in 36 studies. Only one study employed a qualitative approach, while three utilised mixed methods that combined both qualitative and quantitative techniques.

Regarding data collection methods (Figure 5), online surveys were the most frequently employed due to their convenience, cost-effectiveness, and efficiency in gathering large number of responses [82]. Out of the 40 articles, 24 used an online survey to collect data, 10 employed in-person surveys, and 2 conducted telephone surveys. Furthermore, one article used a focus group and another three articles relied on secondary data. In terms of analytical methods employed (Figure 6), 40% of the articles (16 studies) applied multiple analytical methods, whereas the remaining 60% relied on a single analysis approach.

## 3. Results

**Figure 7:**
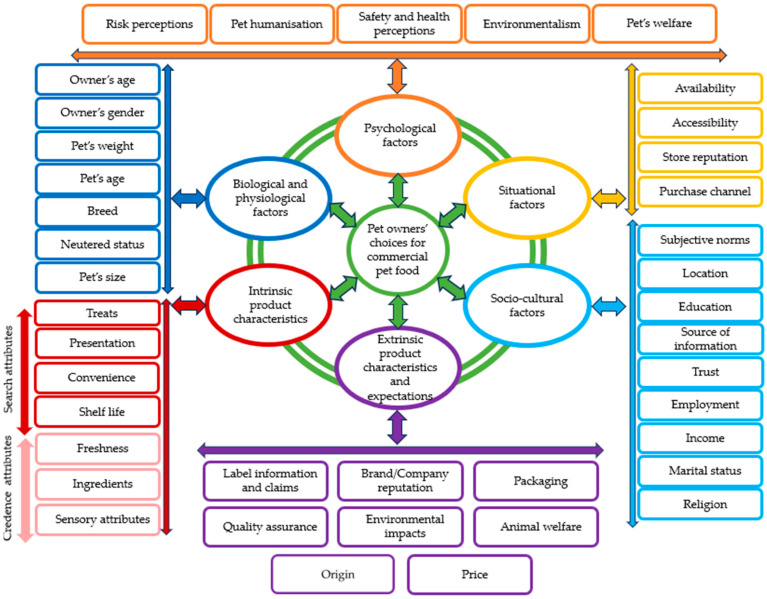
Main drivers of pet owners’ buying behaviours towards commercial pet foods.

This section presents the findings from the reviewed articles using Mojet’s model [83] to organise and display the findings on the drivers of pet owners’ demand for commercial pet food products (see Figure 7). This model categorizes the drivers into six distinct groups or themes: intrinsic product characteristics, extrinsic product characteristics, socio-cultural factors, situational factors, psychological factors, and biological and physiological factors.

### 3.1. Intrinsic Product Characteristics

Similar to human food purchasing behaviour, pet food acquisition is significantly influenced by several sensory factors. These include the product appearance [65,66,80] or presentation [63], colour [63,80], odour [63,80] or smell [65,66], freshness [70,80], and flavour [45] or taste [65,70,80]. Studies focusing on the sensory analysis of dry dog food found product appearance characteristics—such as size, shape, colour, and kibble composition—to be strongly associated with pet owners’ acceptance, although their relative importance varied across countries [48,49,59]. For instance, Polish pet owners preferred products featuring single-kibble designs of medium size, brown colour, and traditional shapes [48]. In contrast, Thai pet owners showed greater interest in yellowish and bone-shaped products [59]. In the US, pet owners placed greater emphasis on colour, favouring products with colour diversity, oily appearance, single kibble’ uniform shape, and low aroma with grain-type notes [49]. Across all three countries, pet owners showed less preference for products with extra-small or large kibbles, multiple sizes, and light or dark colour tones [48,49,59].

Pet food ingredients were also identified as key factors influencing pet owners’ purchasing decisions regarding commercial pet foods. For instance, Di Donfrancesco et al. [75] investigated the acceptance of sorghum-based dog food compared to conventional formulations containing wheat, rice, or maize. Their study evaluated attributes such as appearance, colour, aroma, and overall appeal to both dogs and their owners. The results indicated that sorghum-based diets were well accepted by both groups, underscoring the market potential of sorghum as a primary ingredient in dry dog food. Similarly, the growing popularity of grain-free pet food, which represents more than 40% of all dry dog food products available in the United States [52], further illustrates the importance of ingredients in consumer choices. Banton et al. [52] found that men and individuals who valued convenience over ingredient quality were less likely to purchase grain-free products. Conversely, owners who believed their dogs had food allergies, preferred dietary variety, or personally avoided grains were more inclined to choose grain-free options.

Another ingredient that has attracted considerable research interest and is increasingly included in commercial pet foods is insects, which serve as a sustainable protein alternative to conventional livestock meats [84,85,86]. Insects are rich in high-quality nutrients, including proteins, essential amino acids, fatty acids, lipids, vitamins, and minerals [85,86,87], and have performed well in palatability assessments [85,88,89,90,91]. Recent studies have also indicated that insect-based diets may be hypoallergenic [92] and highly digestible for pets at different life stages, with no adverse effects on physiological health [87,93,94,95,96]. However, consumer acceptance of insect-based pet foods remains mixed. Higa et al. [97], Baptista da Silva et al. [64] and Leriche et al. [98] reported relatively high acceptance levels, whereas Kępińska-Pacelik and Biel [85] observed continued consumer hesitation, primarily due to concerns regarding safety, digestibility, allergic reactions, and potential contamination. Factors such as food technology neophobia and empathy for animals were found to reduce pet owners’ willingness to pay (WTP) for insect-based products, while greater concern for sustainability increased their WTP [69]. These findings highlight the importance of educating consumers on the environmental and health advantages of incorporating insects into pet food formulations [99]. Baptista da Silva et al. [64] reported that both dog and cat owners who have positive entomophagy experience or show interest in entomophagy increase the acceptance of insect-based pet food. Another study by Pinney and Costa-Font [42] pointed out that the pet owners’ intention to try or buy insect-based dog food was positively affected by positive attitude towards insect-based pet food, social norms, perception of benefits of insect consumption, food preference for animal welfare, health and environment, while negatively affected by beliefs regarding insect sentience and perception of risks of insect consumption.

Sustainability, ethical values, and animal welfare concerns have been shown to influence pet owners’ decisions to adopt vegetarian or vegan diets for their pets [100]. Dodd et al. [53] reported that nearly all pets on plant-based diets were owned by vegan individuals. Despite this rising trend, questions remain about the nutritional adequacy of such diets—especially for cats, which are obligatory carnivores requiring nutrients such as taurine, long-chain polyunsaturated fatty acids, and vitamin A, all of which are scarce in plant-based sources [101,102]. In a subsequent study, Dodd et al. [103] compared owner-reported health outcomes in cats fed plant-based versus meat-based diets and found no significant association between diet type and lifespan.

In addition to insect-based and plant-based options, cultivated meat is another ingredient that appeals to pet owners concerned about the ethical implications of animal husbandry [51]. Currently, cultivated meat products for human consumption are predominantly available in North American markets and are rapidly expanding in Asia-Pacific countries [104]. Oven et al. [51] revealed that 81.4% of pet owners open to consuming cultivated meat themselves were also open to feeding it to their pets. Interestingly, vegans and vegetarians were less inclined to eat cultivated meat but more willing to offer it to their pets. Furthermore, those who perceived cultivated meat as unnatural, unsafe or unethical and already satisfied with their pets’ current diets, were less likely to feed their pets cultivated meat. The study also revealed that males pet owners under 40 years of age showed a higher likelihood of feeding their pets cultivated meat, although these effects were only weakly significant. Overall, perceptions of health and safety appear to be key barriers to consumer acceptance of cultivated meat-based pet food products. To promote adoption of cultivated meat-based pet food, pet food manufacturers and marketers should focus on transparent communication regarding its safety and nutritional adequacy.

Quality and healthfulness are also important intrinsic attributes influencing pet food choices [55,65,68,71,80]. Studies revealed that pet owners prioritize nutritional composition and prefer products they perceive as nutritionally complete [45,55,61,63,68,70]. In addition, factors such as convenience and shelf life have been found to significantly influence pet owners’ purchasing decision [55,62,80]. For instance, pet owners are likely to prioritize products that are easy to prepare and store [63,65,70,72,80]. Downes et al. [72] reported that pet owners highly value the longevity and durability of certain products. This factor is especially important when choosing between a dry and wet pet food. There is a higher demand for dry food products over other their wet counterparts due to their ease of storage and convenience [105]. Conversely, wet pet food characterised by its high water and protein content, and low-calorie density were food to appeal more to pet owners who seek to protect their pets from dehydration, reduce the risk of urinary tract infections, and prevent weight gain [106].

In addition to dry and wet food, pet owners regularly purchase treats as a way to strengthen their emotional bond with their pets. Treats can account for up to 15% of a pet’s caloric intake [54,56,107]. However, frequent treat feeding has been identified as a major contributor to the rising prevalence of obesity in cats and dogs, which has become a common and growing pet health concern [107,108,109]. A recent survey reported that 82.7% of dog owners in the US and Canada feed their pets with commercial pet treats and 42% consider treats to be part of their dogs’ regular diet [54]. Similarly, a study by White et al (2016) reported that 69% of UK pet owners revealed to buy commercial treats for their pets daily [56]. Even though treats are a consistently growing segment within the pet food industry, very little attention has been paid to consumers’ buying motivations for this type of pet food products. Recent studies have highlighted that pet owners’ choices of treats are influenced by factors such as ingredients, health claims, brand, production origin, price, and sensory characteristics, including flavour, taste, shape, size, and texture [54,73]. Among them, the ingredients of treats and taste were mostly valued by pet owners, while price was a relatively less important factor [54,73].

### 3.2. Extrinsic Product Characteristics

Previous studies have identified several key extrinsic pet food attributes that pet owners prioritize when selecting commercial pet food products, including brand reputation [45,66,67,70,71,80], company reputation [58], origin [65,66], product claims [52], packaging [65,80], quality of trademark [63], label information [61,66], and quality assurance [45]. Michel et al. [61] found that pet owners’ trust in label information, pet food companies, and manufacturers play an important role in shaping their attitudes towards commercial pet food. Morelli et al. [73] found that 75% of pet owners followed the feeding instructions in the label.

Numerous studies identified price as a key determinant of pet owners’ purchasing decision of commercial pet food products [44,45,52,55,58,63,65,66,70,71,72,80]. For instance, Kwak and Cha [71] found that price fairness was positively associated with positive consumers’ attitudes towards commercial pet food. Schleicher et al. [80] pointed out that pet owners are more likely to buy pet food products if they are on sale. Similarly, Boya et al. [70] and Suarez et al. [63] found some pet owners actively seek low-priced pet food products, special offers or discounts. However, this price sensitivity is not uniform across all pet owner segments. For example, owners with normal-weight dogs tend to place less importance on low prices [63]. Older owners were found to place greater importance on higher priced pet food products than younger owners [66]. In contrast, the growing number of pet owners worldwide who view themselves as ‘pet parents’ look was found be willing to pay premium prices for pet food products with desirable attributes such as organic, made of natural ingredients, no artificial additives, and claims customised for specific lifestyle needs [60,110].

The environmental sustainability cost of pet food production and consumption has been widely examined in several studies [33,35,36,111]. In response, a range of more sustainable pet food products such as insect-based, plant-based, and algae-based pet food has emerged claiming to maintain pets’ good health while minimizing the environmental impact of pet food production and consumption [69,112]. However, consumers acceptance of these novel pet food products remains low due to concerns regarding safety and ethical considerations [113,114,115]. Despite many pet owners revealed to view environmental sustainability as a key determinant of their choices, this positive perception is not translated into actual purchases of more sustainable pet food products including novel product such as plant-based and insect-based pet food [57,69].

Another issue to consider when discussing pet food sustainability is the growing trend of pet humanization. This has led a segment of pet owners to demand pet foods with high-quality ingredients, such as fresh vegetables, and premium cuts of meat [76]. However, this trend has resulted in an increase in food waste as pet food manufacturers often discard ingredients that are safe for consumption but do not meet human food standards. For example, meat near the bones is frequently discarded during the production of pet food with human-grade standards. To address this issue, the concept of upcycling has been introduced as a sustainable solution to reduce food waste from pet food processing. Upcycling transforms food, which would otherwise be discarded (e.g., by-products), into valuable new products such as dog treats [76,116]. In a study that analysed pet owners’ purchase intention for upcycled pet food, Ye et al. [76] found that pet owners reported higher purchase intentions and positive perceptions of quality and sustainability toward upcycled pet food compared to conventional alternatives. However, the positive perceptions diminishes as prices increased.

### 3.3. Situational Factors

In addition to product attributes, situational factors such as product availability and accessibility have been reported as essential factors for pet owners [44,77]. Many pet owners prioritise purchasing convenience [58,77,117], often opting to buy pet food at the nearest supermarket [63]. Additionally, the type and reputation of the store also play a crucial role in where pet owners decide to shop [67,70]. Pet food is typically purchased through three main sales channels: online, pet stores and retailers [77]. Among these, some pet owners prefer shopping online, where store reputation serves as a key indicator of product quality [67]. In contrast, others favour in-store purchases, valuing the contact of interaction as part of their shopping enjoyment [70,77]. Stoica and Hickman [81] found that for Gen Z pet owners, social media engagement was the key determinant of their purchase intention in e-commerce and social media commerce. In contrast, they purchase in-store when seeking professional advice.

An emerging purchase channel is subscription box retailing. It is an e-commerce business model where consumers receive periodic deliveries of customised product boxes after paying a subscription fee [118]. The custom nature of these online subscription services has contributed to the massive growth of the personalised product economy in recent decades [119,120]. Based on PLS-SEM, Lima et al. [68] found that customer satisfaction, perceived healthfulness, ingredients’ nutritional composition and product extrinsic attributes played the core role in consumers’ continued intention to use subscription-based online services for pet food. The relationship between price and continuance intention was found to be insignificant, indicating that customers have strong loyalty and perceive significant value in the service that surpasses their concerns about cost.

### 3.4. Sociocultural Factors

Besides the context and the attributes of the products, socioeconomic and cultural factors have also been reported as important in driving pet owners’ purchasing decisions. These elements are, among others, employment [44,66], education [47,66,67], income [47,60,79], budget [67], marital status [79] and the resident location [66,67,79]. Naughton et al. [44] found that female cat owners in non-animal-related jobs prioritise their pets’ preferences in food purchases, while those in veterinary professions focus more on health benefits. They also found, as in Rombach and Dean [47], owners with higher education levels placed less emphasis on price and did not limit their pet food purchases to what was available in the store. In line with these findings, Xiao et al. [67] observed that Chinese pet owners with higher education levels are more attentive to brand reputation when shopping online. Kumcu and Woolverton [60] reported that owners with higher education, those with higher income, and those with fewer household members are more likely to purchase premium pet food.

Geographical location has also been shown to influence pet owners’ purchasing preferences. Xiao et al. [67] reported that pet owners residing in Beijing place greater importance on product ingredients compared to those in less populated areas of China, likely due to the higher income and education levels typically found among Beijing residents. Similarly, in Romania, Cozma et al. [79] observed that pet owners living in major cities resumed in-person purchasing more quickly than those in other regions during the post-COVID period.

Religious beliefs have been identified as relevant in shaping pet owners’ purchase intentions for pet food in Indonesia. Prawira and Pangaribuan [78] using SEM found that religious cues on packaging directly enhance purchase intention and that pet attachment and religion can indirectly affect purchase intentions by positively influencing the presence of religious cues on the packaging. However, it remains uncertain whether this trend also applies to other religious countries, indicating a need for further investigation.

Information has been identified as an important factor in shaping pets’ diet-related decisions. Conway and Saker [58] compared pet owners’ willingness to feed grain-free food before and after reading an informational brochure on environmental sustainability and grain-free diets. They found that educational brochures can effectively enhance owners’ knowledge and ability to identify more sustainable diets, leading to a decrease in feeding grain-free diets to their pets. These findings highlight the importance of public education in raising pet owners’ awareness of the environmental and health implications of their pets’ diets. Another example illustrating the focus on nutritional quality and misalignment between sustainable and healthy diets is the trendy high-protein ‘ancestor diets’ advertised by pet food companies as healthier alternatives to grain-inclusive pet foods. These pet diets compromise environmental sustainability as they encourage the excessive use of animal-based ingredients while minimising the use of more sustainable plant-based ingredients and animal by-products [121].

The source of information that consumers rely on to support their decisions has been evaluated by several articles, which reported that many pet owners rely on recommendations from veterinarians [54,55,63], food preparation experts and health care professionals [70] to provide their pets with healthier diets. A study by Nielson et al. [54] highlights that younger pet owners tend to prioritise expert advice over online information or suggestions from family and friends when selecting products. Despite this reliance on veterinary expertise, some pet owners may still disagree with veterinarians on issues like body condition [122,123,124] and weight management [125]. Additionally, some articles reported that pet owners seek product information directly from pet store staff [52,54], from their friends, family members, co-workers [54,70] and other experienced pet owners [63]. This behaviour indicates that while objective veterinary advice serves as a guiding factor for pet owners, subjective norms—defined as the social expectations that influence an individual’s perception of whether to engage in a particular behaviour—may also significantly shape pet owners’ purchasing intentions [126].

In addition, social media has emerged as a critical source of information, shaping consumers’ decision-making processes [127]. Previous studies pointed out that pet owners use online reviews and recommendations to assess the quality of various substitute products and make their final purchasing decisions [52,54]. Influencers and online reviewers play a key role in creating content, such as product endorsements and evaluations, that help companies enhance their brand image and promote their products to a broader audience, ultimately boosting consumer purchase intentions [127]. This is especially the case for Gen Z pet owners [81]. However, older generations, especially baby boomers, comprise a significant portion of pet owners (e.g., 24% in the U.S.) [128], may struggle with digital technology, distinguishing between real and fake information, and identifying online scams, leading them to trust traditional information sources more than online ones [129]. As a result, social media may have limited influence on their purchasing decisions.

Another source of information reported in the literature is advertising. This remains another powerful marketing strategy for companies to communicate product information to target consumers. Through compelling words, visuals, and imagery, advertisements are designed to capture consumers’ attention and persuade them to choose their products over those of competitors [130]. The effectiveness of advertising is also evident in the pet food market, where it serves as a key source of information for pet owners regarding a product’s functions, properties, brands, and special offers. As a result, advertisements become a crucial aspect to consider in their decision-making process when purchasing pet food [63,70].

### 3.5. Psychological Factors

Pet owners’ perceptions—which include their opinions, beliefs, emotions, feelings, and expectations surrounding all aspects of pet ownership [131]—have been shown to significantly influence their pet food choices. The growing trends of pet humanisation and anthropomorphism have played a major role in shaping both pet-related marketing strategies and consumer behaviour [117]. Many owners have developed a strong emotional attachment to their pets, often viewing themselves as “pet parents” who prioritise their animals’ health and wellbeing, sometimes even above their own [43,132]. Boya et al. [70] categorised U.S. dog owners into distinct market segments based on key dimensions of anthropomorphism and dog-oriented self-concept: (i) Dog people—highly involved with their dogs, treating them as human companions. (ii) Dog parents—less likely to define themselves through their dogs but still regard them as family members, similar to children. (iii) Dog owners—consider dogs part of the family but primarily as pets, treating them differently from children. Significant differences emerged in dog food choice criteria across these groups. Dog people placed the greatest emphasis on health, nutrition, quality, freshness, taste, and dietary variety, indicating a strong concern for their pets’ overall feeding experience. Similarly, dog parents also prioritised health, nutrition, quality, and freshness, though to a lesser extent. These groups have shifted away from valuing only price and convenience toward prioritising product quality, a trend that has contributed to the growth of the premium pet food market [60,110,133]. In contrast, dog owners assigned lower importance to most choice factors except for price and store type, indicating a preference for purchasing discounted products or shopping at grocery stores for convenience. They tend to view pets primarily as animals with basic needs, which makes them more price-sensitive [117].

Boya et al. [70] also compared consumers’ human food choice criteria with pet food choices, finding that only dog people place less importance on price when buying food for their dogs compared to when purchasing food for themselves. This shows they prioritise their dogs’ food’s healthfulness and nutritional value more than their own diets, and they are also more likely to be loyal to specific pet food brands. Furthermore, dog people prioritise the information provided by healthcare professionals, with less trust in advertising and social media [70]. Dog-oriented self-concept was also considered by Rombach and Dean [46] who considered to what extent it is a determinant of pet food anxiety and change in pet food shopping behaviour in the case of a disruption of the pet food supply chain, such as during the COVID pandemic. In this context, pet food anxiety is understood as the anxiety generated by pet food shortages and the consequence of the inability to feed pets the same way they are used to without disruption. Results show that all groups were likely to experience pet food anxiety during the supply chain disruption, whether they perceived pets as family or just animals. Furthermore, owners with an increase in pet food anxiety were also revealed to be likely to exhibit changed shopping and feeding behaviour. Overall, Boya et al. [70] pointed out a clear need for pet food marketers to divide pet owners into different marketing segments rather than treating them as a single, uniform group of buyers. However, Rombach and Dean [46] showed that a lack of pet humanisation behaviours doesn’t mean a lower level of care for pets or worry about their feeding needs.

A further aspect of pet owners’ perceptions that influences the choice of pet food is health and risk perceptions. Eagan et al. [57] found that pet food safety is an important concern for pet owners, especially regarding the pathogens in the products. Many owners follow pet food handling practices such as washing hands after handling raw food or feeding their pets and storing the pet food away from human food to reduce the potential health risks. Furthermore, Michel et al. [61] found that some pet owners choose not to feed their pets with commercial pet food since they don’t trust the safety of food additives and consider that additives in commercial pet food can have side effects on pets’ health. Although many world health authorities claim that food additives are essential in commercial pet food and must meet safety requirements before being put into food manufacturing [134,135,136], many pet owners decline to use commercial pet food, showing significant conflicts between owners’ beliefs and the authorities’ assurances about this issue.

Regarding environmental sustainability, Conway and Saker [58] found that pet owners considered other factors such as health, cost, ingredients, nutrition, and veterinarian recommendations over environmental sustainability when deciding their pets’ diet. Suarez et al. [63] found that both pet owners with normal and overweight dogs tended not to be concerned if the food package was environmentally friendly. Similarly, in the study of Eagan et al. [57], pet owners agreed on the definition of environmental sustainability and admitted that this idea was important to them. However, sustainability did not motivate their food purchasing choice. This discrepancy suggests that, for many pet owners, environmental sustainability remains an abstract concept that does not translate into tangible benefits in their daily lives. Therefore, it is crucial for society and pet food manufacturers to work towards achieving sustainable development goals and help reshape pet owners’ perceptions and preferences for environmental sustainability.

Apart from physiological factors, some articles emphasize that pet welfare is another important factor in owners’ food choices. Those products that pets show affection or preference for would be primarily selected by their owners [44,63,66,80]. Apart from the food preference, pet owners are also seeking products that can cater for the specific health needs of their pets. Morgan et al. [55] found that pet owners primarily feed their pets with diets that can solve existing health issues, benefit dental health and body conditions, as well as produce consistent stool. Similar findings were found by Vinassa et al. [66], as they pointed out that Italian pet owners would consider whether their pets can have normal stool appearance and shiny coats as important indicators to assess the quality of pet food products. These findings reflect that pet owners care about their pets’ quality of life, not their basic living needs. In the same line, White et al. [56] revealed that some owners gave treats to their dogs to make them feel happy. These owners felt that not feeding dogs with treats was like not giving kids toys. Other owners regarded treats as a welcome addition to boring diets.

### 3.6. Biological and Physiological Factors

Previous studies have identified age [44,47,52,66,67] and gender [44,67] as influential factors shaping pet owners’ purchasing patterns for pet food. Xiao et al. [67] reported that younger pet owners place greater importance on brand reputation, while older Chinese consumers are less likely to pay attention to ingredient information when shopping online and often do not read product labels. Similarly, Vinassa et al. [66] found that pet owners over 65 tend to prioritise price over factors such as feed appearance or animal satisfaction, whereas those under 35 focused more on stool quality, protein content, and eco-friendly packaging. Rombach and Dean [47] further observed that older pet owners are generally less interested in the convenience aspect of pet food products. In addition to age, gender has also been shown to influence pet food purchasing behaviour. Xiao et al. [67] found that men are generally less concerned about the reputation of pet food retailers compared to women. Conversely, other research conducted in the United States reported that consumers’ acceptance of dry dog food was not significantly associated with demographic variables such as income, age, gender, or education, suggesting that these factors may not consistently determine individual preferences [49].

Recent studies have underscored the significance of pets’ physiological status as a key determinant in consumers’ pet food choices. Prata [45] reported that pet owners tend to prefer products specifically formulated to match their pets’ metabolic needs and age. Additionally, when purchasing dog food, many owners consider factors such as size [65,67], breed, age, and neutered status [55] to ensure that the products selected appropriately meet their pets’ nutritional requirements. Body weight has also been identified as an important factor influencing purchasing behaviour. Owners of normal-weight dogs tend to place greater emphasis on product presentation, ingredient quality, and nutritional composition, whereas those with overweight dogs are more likely to prioritise low-cost options or products on special offer [63].

## 4. Discussion

This study investigates the factors influencing pet owners’ decisions and behaviours in purchasing commercial pet food. It consolidates the current state of knowledge, identifies key research gaps, and proposes directions for future studies that aim to deepen the understanding of consumer demand in this evolving market.

### 4.1. Key Drivers of Consumer Behaviour

The findings of this review suggest that pet owners’ purchasing behaviour for commercial pet food products is influenced by factors comparable to those that shape human food choices [137,138,139]. This supports the applicability of Mojet’s model, originally developed to explain human food consumption, as an appropriate framework for understanding the decision-making processes underlying pet food purchasing behaviour.

**Figure 8:**
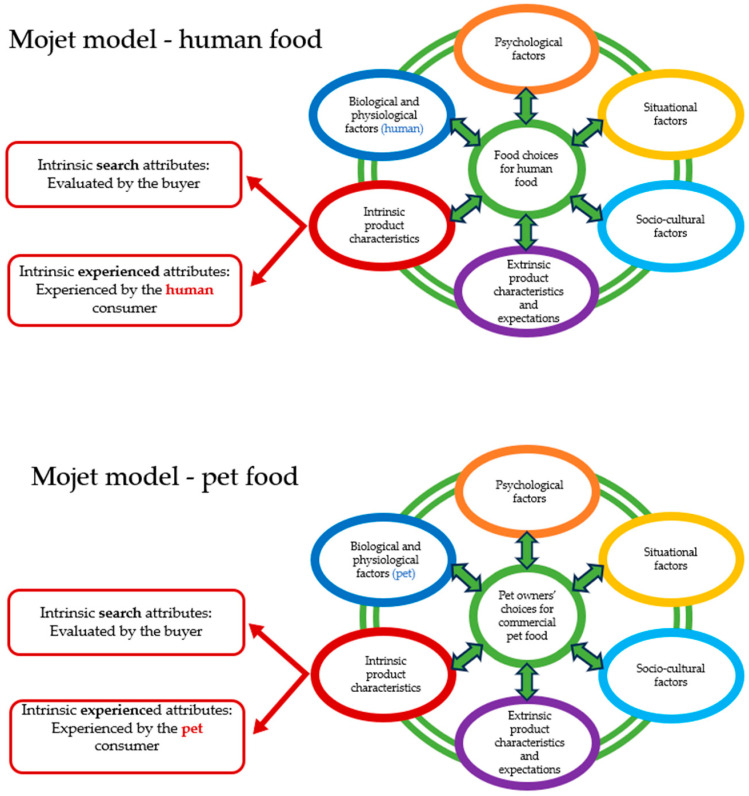
Comparison of feedback pathways in human and pet food purchase decision-making.

However, an important distinction between Mojet’s model for human food and its application to pet food lies in the nature of certain intrinsic attributes. In the case of pet food, intrinsic characteristics such as taste, flavour, freshness, and ingredients are largely credence attributes—qualities that pet owners cannot directly experience (see Figure 8). Consequently, owners often rely on external cues to infer these attributes, such as sensory descriptions on packaging (e.g., “freshly cooked scent,” “crunchy kibble,” “meaty flavour”) or their pets’ body language while eating (e.g., signs of enjoyment such as running eagerly to the food bowl, eating immediately, or maintaining focus on the dish). In addition to their own observations and judgments, pet owners also depend on socio-cultural factors, including trust, available information, and subjective norms, when forming their purchase decisions. This reliance may explain why veterinarians emerge as key intermediaries between consumers and the pet food industry, serving as trusted advisors on nutrition, health, and product selection [140,141]. Their role is particularly crucial in encouraging the adoption of sustainable and health-oriented diets, such as insect- or plant-based foods, which are often met with consumer hesitation due to unfamiliarity or perceived safety concerns [57,69,112]. Future research should therefore examine how veterinarians’ recommendations and packaging elements (e.g., sensory descriptions) influence pet owners’ willingness to adopt sustainable pet food options and reduce dependence on low-quality treats.

This literature review also revealed that the majority of existing studies have focused on dry dog food, leaving a notable research gap concerning wet food and commercial products for other pet species (e.g., rabbits, fish, and birds). Future research should therefore explore the factors influencing pet owners’ demand for food intended for these less-studied species and examine how these drivers compare to those shaping the demand for dog and cat food products. Such investigations would provide a more comprehensive understanding of consumer behaviour across the broader pet food market.

Compared with the extensive literature on human food, research examining the impact of market and policy interventions, such as taxes, subsidies, eco-labels, nutritional claims, advertising, price promotions, and information campaigns, on pet food purchasing behaviour remains very limited. Given the demonstrated effectiveness of these interventions in influencing consumer behaviour within the human food sector, future studies should prioritise evaluating the potential of fiscal and information-based policies to encourage the demand for pet foods whose production and consumption can reduce the environmental footprint of the pet food industry while simultaneously improving animal health and welfare.

The results of this review also indicated that pet owners’ socio-cultural background, self-perception, age, gender, and professional affiliation (e.g., veterinary training) significantly influence their preferences for commercial pet foods [44,66,70]. This suggests that pet owners’ preferences are highly heterogeneous, and the reliance of most studies on average preference estimates may obscure substantial variation across different consumer groups. Future research on pet owners’ preferences and willingness to pay for commercial pet food products should therefore place greater emphasis on examining preference heterogeneity among distinct segments of pet owners, e.g., using a latent class modelling approach to assess the heterogeneity of preferences. Understanding these differences is essential for marketers and policymakers to design market and policy interventions better tailored to the diverse needs, values, and motivations of various pet owner groups.

Finally, the results indicate that several desirable pet food attributes, such as being organic, healthier, or locally produced, are highly valued by pet owners. However, this may create a misleading impression for marketers and policymakers that incorporating multiple desirable attributes will generate a simple additive effect, where the overall value of a product bundle equals the sum of the individual attribute values. This assumption holds only if pet owners perceive these attributes as independent. Nonetheless, if attributes are perceived as complements or substitutes, the total value of the bundle may be higher (in the case of complementarity) or lower (in the case of substitutability) than the sum of individual attribute values. To address this issue, future research should go beyond estimating the isolated effects of individual attributes and explicitly account for potential interactions between desirable attributes when modelling pet owners’ preferences and valuations

### 4.2. Research Context

Existing studies on consumer behaviour in the pet food sector have focused primarily on dog and cat food, revealing a significant gap in research on other companion animals such as fish, rabbits, birds, and turtles—species that are increasingly common in households worldwide. For instance, freshwater fish are now the third most common household pet in the United States, with an estimated 11.1 million households owning them [128]. Similar trends are evident in the United Kingdom and Spain, where aquaria and domestic bird populations rival or even surpass those of cats [1]. These developments highlight an evolving market landscape with untapped potential for diversification and innovation in non-traditional pet food segments.

Geographically, most studies have examined European and North American markets, while emerging economies in Africa, Asia, and South America remain largely unexplored despite rapid growth in pet ownership and rising disposable incomes [1,5]. Understanding the cultural and economic nuances of these markets could offer valuable insights for companies expanding their global presence. Given the observed heterogeneity in consumer behaviour across countries [52], cross-national comparative studies are strongly recommended to explore how cultural, socioeconomic, and policy contexts shape purchasing decisions.

### 4.3. Theoretical and Methodological Considerations

The review reveals a general lack of theoretical grounding in existing research, with only a few studies explicitly applying consumer behaviour theories such as the Theory of Planned Behaviour (TPB), Self-Medication Effect (SME), Affective Response Theory (ART), or the Three-Circle Analysis Framework. Future studies should extend existing theoretical models—such as TPB—or incorporate alternative frameworks including Control Theory [142], Health Action Process Approach [143], Protection Motivation Theory [144], Attribution Theory [145], Self-Determination Theory [146], and Social Influence Theory [147]. Such integration would provide a richer, more nuanced understanding of the complex psychological mechanisms underlying pet food choices.

From a methodological standpoint, research in this field remains heavily quantitative, relying predominantly on surveys. While quantitative approaches offer advantages such as transparency, scalability, and statistical robustness, they often overlook the depth and contextual richness of consumer motivations. Future research should incorporate qualitative and mixed-method designs, including interviews, focus groups, and ethnographic approaches, to explore the underlying beliefs, emotions, and values that shape pet owners’ purchasing behaviours. Group-based techniques, particularly focus groups or anonymous online discussions, could also help capture behavioural heterogeneity while reducing response bias [40,148].

Emerging digital research methods such as netnography are especially promising, as they leverage social media and online reviews to analyse real-world consumer attitudes [149,150]. The increasing use of e-commerce and online subscription services in the pet food industry [65,68] suggests that netnography could become a vital methodological tool for understanding evolving consumer preferences.

In terms of data analysis, existing studies have mainly relied on descriptive statistics and basic correlation analyses. More sophisticated techniques such as structural equation modelling (SEM), regression modelling, and econometric approaches (e.g., Cox survival models, Almost Ideal Demand Systems, and choice modelling) could help explain the causal relationships among key variables and simulate real-world market conditions [151,152,153,154]. Additionally, augmented reality (AR) technologies, which enhance online purchasing experiences by enabling virtual product interaction, offer a new avenue for studying how visual engagement affects pet owners’ WTP [155,156].

### 4.4. Limitations and Future Directions

This SLR has several limitations that should be acknowledged and considered in future reviews within this domain. First, the review was restricted to peer-reviewed journal articles, book chapters, and technical reports written in English, which may have led to the omission of relevant research published in other languages. Future reviews are encouraged to expand their search to include non-English relevant publications to reduce the likelihood of missing important evidence and enhance the literature search’s comprehensiveness and robustness.

Second, to maintain a clear distinction between pet food and human food products, this review excluded raw meat, homemade diets, and other non-commercial pet foods, as these products often overlap with human food markets. Expanding future reviews to include such non-commercial feeding practices could yield valuable insights into pet owners’ motivations, attitudes, and psychological factors influencing their choices between commercial and non-commercial pet food options.

Despite these limitations, this SLR contributes to a still limited body of literature on consumer behaviour toward pet food. It provides a comprehensive overview of existing evidence, highlights key research gaps, and proposes clear directions for future inquiry. As such, it is expected to serve as a foundation for subsequent academic research in this growing field.

## 5. Conclusions

This systematic review provides the first comprehensive synthesis of the drivers influencing pet owners’ purchasing behaviour for commercial pet food. It identifies six key categories of determinants and underscores the need for theory-driven, interdisciplinary, and methodologically diverse research approaches. While the existing evidence demonstrates that pet food choice is shaped by a complex interplay of psychological, product-related, and socio-cultural factors, the literature remains fragmented and overly focused on specific species and regions. Future research should integrate insights from marketing, behavioural economics, veterinary science, and environmental psychology to develop a holistic understanding of consumer behaviour in the pet food sector. By bridging these disciplinary divides, scholars can contribute to building a sustainable, evidence-based pet food system that benefits pets, owners, and the environment alike.

## Figures and Tables

**Table 1:** Selected keywords and search string.

**Keywords related to commercial pet food**	“pet food”, “commercial pet food”, “dog food”, “cat food”, “insect pet food”, “commercial cat food”, “commercial dog food”, “dry pet food”, “wet pet food”, “sustainable pet food”, “pet fish food”, “ornamental fish food”, “commercial pet fish food”, “pet rabbit food”, “commercial rabbit food”, “pet bird food”, “commercial pet bird food”.
**Keywords related to consumer behaviour**	“pet owner perceptions”, “owner perception”, “pet owner behaviour”, “pet owner food choice”, “pet food choice”, “pet owner purchase intention”, “consumer perception”, “consumer behaviour”, “consumer purchase intention”, “consumer food choice”, “attitude”, “factors”, “attributes”, “demand”, “purchase”, “pet food preference”, “food preference”, “purchase intention”, “buying behaviour”, “consumer buying behaviour”, “buying pet food”, “buying dog food”, “buying cat food”, “consumer acceptance”, “pet food purchasing”
**Search string**	(“pet food” OR “commercial pet food” OR “dog food” OR “cat food” OR “insect pet food” OR “commercial cat food” OR “commercial dog food” OR “pet fish food” OR “ornamental fish food” OR “ commercial pet fish food” OR “pet rabbit food” OR “commercial rabbit food” OR “pet bird food” OR “commercial pet bird food” OR “dry pet food” OR “wet pet food” OR “sustainable pet food”) AND (“pet owner perceptions” OR “owner perception” OR “pet owner behaviour” OR “pet owner food choice” OR “pet food choice” OR “pet owner purchase intention” OR “consumer perception” OR “consumer behaviour” OR “consumer purchase intention” OR “consumer food choice” OR “attitude” OR “factors” OR “attributes” OR “demand” OR “purchase” OR “pet food preference” OR “food preference” OR “purchase intention” OR “buying behaviour” OR “consumer buying behaviour” OR “buying pet food” OR “buying dog food” OR “buying cat food” OR “consumer acceptance” OR “pet food purchasing”)

**Table 2:** Eligibility criteria in the article selection process.

Inclusion Criteria	Exclusion Criteria
Available online	Unavailable
Studies focusing on commercial pet food or commercial pet food were part of the study focus	Studies on raw meat, homemade food, table scraps or leftovers and other non-commercial pet food.
Studies were to analyse consumer behaviour towards commercial pet food, for example, consumers’ buying behaviour, decision on food choice etc.	Studies not related to consumer behaviour research
Peer-reviewed journal articles, peer-reviewed book chapters, technical reports	Non-peer-reviewed journal articles, conference papers, market reports, news, magazine articles, Master/PhD thesis, white papers, and papers published in a predatory or controversial journal
Written in English	Written in other languages

**Table 3:** Summary of journal sources, research countries and research methodologies of included articles.

Journal Title	Number of Articles	Research Methods	Data Collection Methods	Research Countries	Authors
*Animals*	**8**	Quantitative	Online survey	The UK	Pinney and Costa-Font [42]
		Quantitative	Online survey	The USA	Tu et al. [43]
		Quantitative	In-person survey	The UK	Naughton et al. [44]
		Quantitative	Online survey	Portugal	Prata [45]
		Quantitative	Online survey	The USA	Rombach and Dean [46]
		Quantitative	Online survey	The USA	Rombach and Dean [47]
		Quantitative	In-person survey	Poland	Baquero et al. [48]
		Quantitative	In-person survey	The USA	di Donfrancesco et al. [49]
*PLoS ONE*	4	Quantitative	Online survey	Brazil	Pedrinelli et al. [50]
		Quantitative	Online survey	The UK	Oven et al. [51]
		Quantitative	Online survey	Cross country	Banton et al. [52]
		Quantitative	Online survey	Cross country	Dodd et al. [53]
*Preventive Veterinary Medicine*	3	Quantitative	Online survey	Cross country	Nielson et al. [54]
		Mixed	Online survey	The UK	Morgan et al. [55]
		Mixed	In-person survey	The UK	White et al. [56]
*Frontiers in Veterinary Science*	2	Quantitative	Online survey	Canada	Eagan et al. [57]
		Quantitative	In-person survey	The USA	Conway and Saker [58]
*Journal of Food Products Marketing*	2	Quantitative	In-person survey	Thailand	Koppel et al. [59]
		Quantitative	Secondary data analysis	The USA	Kumcu and Woolverton [60]
*Journal of the American Veterinary Medical Association*	2	Quantitative	Telephone survey	Cross country	Michel et al. [61]
		Quantitative	Telephone survey	The USA	Wakefield et al. [62]
*Journal of Animal Physiology and Animal Nutrition*	2	Quantitative	In-person survey	Spain	Suarez et al. [63]
		Quantitative	Online survey	Belgium	Baptista da Silva et al. [64]
*Agribusiness*	1	Mixed	Secondary data analysis	The USA	Hobbs et al. [65]
*BMC Veterinary Research*	1	Quantitative	In-person survey	Italy	Vinassa et al. [66]
*Chinese Economy*	1	Quantitative	Online survey	China	Xiao et al. [67]
*Electronic Commerce Research*	1	Quantitative	Secondary data analysis	Not specified	Lima et al. [68]
*Food Research International*	1	Quantitative	Online survey	Italy	Fantechi et al. [69]
*International Journal of Consumer Studies*	1	Quantitative	Online survey	The USA	Boya et al. [70]
*International Journal of Food Properties*	1	Quantitative	In-person survey	Korea	Kwak and Cha [71]
*Irish Veterinary Journal*	1	Qualitative	Focus group interviews	Ireland	Downes et al., 2017 [72]
*Journal of Applied Animal Welfare Science*	1	Quantitative	Online survey	Italy	Morelli et al. [73]
*Journal of Asia-Pacific Entomology*	1	Quantitative	Online survey	Not specified	Siddiqui et al. [74]
*Journal of Cereal Science*	1	Quantitative	In-person survey	The USA	di Donfrancesco et al. [75]
*Journal of Cleaner Production*	1	Quantitative	Online survey	The USA	Ye et al. [76]
*Journal of Multi-Criteria Decision Analysis*	1	Quantitative	Online survey	The USA	Salmani and Partovi [77]
*Multidisciplinary Science Journal*	1	Quantitative	Online survey	Indonesia	Prawira and Pangaribuan [78]
*Springer Proceedings in Business and Economics*	1	Quantitative	Online survey	Romania	Cozma et al. [79]
*The Canadian Veterinary Journal*	1	Quantitative	Online survey	The USA	Schleicher et al. [80]
*Young Consumers*	1	Quantitative	Online survey	The USA	Stoica and Hickman [81]

## Data Availability

Data are contained within the article.
